# Serum miR-210 Contributes to Tumor Detection, Stage Prediction and Dynamic Surveillance in Patients with Bladder Cancer

**DOI:** 10.1371/journal.pone.0135168

**Published:** 2015-08-07

**Authors:** Yongmei Yang, Ailin Qu, Jingkang Liu, Rui Wang, Yingjie Liu, Gang Li, Weili Duan, Qian Fang, Xiumei Jiang, Lili Wang, Guixi Zheng, Lutao Du, Xin Zhang, Chuanxin Wang

**Affiliations:** 1 Department of Clinical Laboratory, Qilu Hospital, Shandong University, Jinan, Shandong Province, China; 2 Department of Urology, Honggang Hospital, Dongying, Shandong Province, China; Saint Louis University, UNITED STATES

## Abstract

MiR-210 is the master hypoxamir that generally exhibits oncogenic properties in most human solid tumors including bladder cancer (BC). However, it remains unknown about the clinical significance of circulating miR-210 levels in BC. In this study, we found that serum miR-210 was up-regulated in patients with BC, and serum levels of miR-210 increased with advancing stage and grade. Moreover, serum miR-210 expression was found to be significantly reduced in paired post-operative samples and elevated in most patients with relapsed BC. Taken together, our data suggest that serum miR-210 could be a potential noninvasive biomarker for screening, predicting and monitoring BC.

## Introduction

Bladder cancer (BC), the most common malignancy of the urinary tract worldwide, results in significant morbidity and mortality [1]. At initial diagnosis, about 70% of BC patients have cancers confined to the epithelium or subepithelial connective tissue (non-muscle-invasive bladder cancer, NMIBC, consist of stages Ta, Tis and T1). More than 50% of these cancers recur and 15–20% progress to muscle-invasive form (MIBC, stages T2, T3 and T4) during follow up [2]. Surely this high rate of disease recurrence requires lifelong surveillance. Currently, the most commonly used test for non-invasive detection of BC is urine cytology. However, this test has limited sensitivity, especially for the detection of low-grade lesions [3–6]. Cystoscopy-guided biopsy for histological evaluation can provide high diagnostic accuracy, but it is invasive and expensive, and inconvenient for general cancer screening. Therefore, there is a need for new markers that may help in non-invasive detection and surveillance of BC.

MicroRNAs (miRNAs, miRs) are a novel class of endogenous, small, non-coding RNA oligonucleotides that could negatively regulate gene expression by targeting the 3’ untranslated region (3’-UTR) of the corresponding mRNA [7]. Cumulative evidence suggests that miRNAs are frequently dysregulated in human cancers and exert significant effects on cancer development [8, 9]. Circulating microRNAs, originate from primary tumor tissues, are stably detectable in serum/plasma [10–12]. Several studies have demonstrated that circulating miRNAs may be used as novel biomarkers in cancers [13–15]. Recently, some circulating microRNAs, such as miR-21, miR-221, miR-375, etc. have been proposed as biomarkers for tumor presence or treatment response to local and systemic therapies [9, 16]. However, the use of circulating miRNAs as blood-based, minimally invasive biomarkers for patients with BC is still relatively less explored.

MiR-210, the master hypoxamir, is upregulated in most human solid tumors and generally exhibits oncogenic properties [17, 18]. Recent studies showed that miR-210 was upregulated in BC tissues[19, 20], and the overexpression of miR-210 was associated with a poor survival, promoted cell growth and migration, and inhibited apoptosis of BC cells [21]. The expression and clinical significance of circulating miR-210 in BC remains unclear at this time. It has been demonstrated that high circulating miR-210 could serve as a biomarker of tumor presence and treatment response to drug therapies in patients with breast cancer [22]. In light of these observations, we postulate miR-210 might be a candidate for exploration as circulating biomarkers of BC and may provide predictive information for improving cancer treatment.

In this study, we focused our analysis on the clinical impact of circulating miR-210 on detection and surveillance of BC, with the hypothesis that circulating miR-210 could serve as a minimally invasive biomarker for BC patients. We found that pre-operative miR-210 expression levels in serum are useful to detect BC, predict muscle invasion and histological degree, and reflect tumour dynamics.

## Materials and Methods

### Patients and samples

The study included 168 patients with newly diagnosed BC in the Department of Urologic Surgery, Qilu Hospital, Shandong University (Jinan, China) between 2006 and 2010. Histological specimens from all patients were reviewed to confirm the diagnosis of BC, and the tumors were staged and graded according to the 2002 TNM classification and the 1973 WHO grading system, respectively. Blood were collected from 168 BC patients before surgery. Paired post-operative blood samples were collected from 40 patients one month after surgery, and unpaired relapse group blood samples were collected from 30 patients. The clinicopathological data of patients, including sex, age, histological differentiation and tumor stage were collected retrospectively. In addition, 104 healthy controls who visited the hospital for a medical check-up were enrolled in this study. Normal controls were selected with similar age and gender proportions to the pre-operative cancer patients, and they were screened to ensure that they were within the normal range of all laboratory findings and had no history of cancer. The collection and analysis of all samples were approved by the Ethical Committee of Qilu Hospital, Shandong University, and written informed consent was obtained from each patient and controls.

Whole blood samples were collected from controls and patients by venipuncture. Serum was separated within 1h of blood collection by centrifugation at 10,000 rpm for 10 min, to completely remove cell debris. The serum samples were then transferred to RNase/DNase-free tubes and stored at -80°C until further analysis. Formalin-fixed paraffin-embedded (FFPE) tissue samples were collected from patients who have underwent radical cystectomy. All FFPE samples were stored at room temperature until use.

### Cell culture

BC cell lines (5637 and T24) and a human uroepithelial cell line (SV-HUC-1) were purchased from American Type Culture Collection (ATCC, Manassas, VA, USA). 5637 and T24 were cultured in RMPI1640 medium containing 10% fetal bovine serum (FBS; Gibco, Carlsbad, CA), whereas SV-HUC-1 was cultured in Dulbecco’s modified Eagle’s medium/F12 (Invitrogen, Carlsbad, CA, USA). All the cell lines were incubated at 37°C in a humidified atmosphere containing 5% carbon dioxide.

### Protocol for the detection of miRNAs in serum

The cDNA of miRNAs in serum was synthesized with the One Step PrimeScript miRNA cDNA Synthesis Kit (Takara Bio Inc., Shiga, Japan). The reaction mixture (20μl) contained 10μl of 2×miRNA Reaction Buffer Mix, 2μl of miRNA Primescript RT Enzyme Mix, 2μl of 0.1% BSA and 3μl of serum mixed with 3μl of serum buffer (2.5% Tween 20, 50 mmol/L Tris and 1mmol/L EDTA [23], and was incubated at 37°C for 60min, at 85°C for 5s and then at 4°C for 60min. After centrifugation at 10 000 rpm for 10 min at 4°C, the cDNA was stored at -20°C until use.

The qRT-PCR for miRNAs was performed in final volumes of 25μl using the SYBR PrimeScript miRNA qPCR Kit (Takara Bio Inc., Shiga, Japan) on ABI PRISM 7500 Sequence Detection System (Applied Biosystems, Foster City, CA). The reaction reagents contained 12.5μl of SYBR Premix Ex Taq II, 0.5μl of DyeII, 2μl of 5μM forward primer (Ribobio, Guangzhou, China), 1μl of 10μM Uni-miR qPCR primer and 2.0μl of template cDNA. The volume was adjusted with RNA-free H2O. The reverse-transcription reaction was performed in triplicate to remove any outliers. According to our previous research, the combination of miR-16-5p and miR-193a-5p were used as the reference gene [24]. And, miRNA expression was recorded as the ratio against the geometric mean of two reference genes. In addition, we selected the minimum miR expression sample and set the expression value of this sample as 1. The expression levels of all the other samples were equal to the relative ratio.

### Isolation of RNA, cDNA synthesis and quantitative real-time polymerase chain reaction (qRT-PCR)

Total RNA extraction from cells and culture media samples were performed using TRIzol reagent (Ambion, Life Technologies, Carlsbad, CA, USA), whereas RNA extraction from Formalin-fixed and paraffin-embedded (FFPE) samples was performed using the miRNA isolation Kits (Bioteke, Beijing, China). All of the manipulations of the RNA were carried out under RNase-free conditions. The isolated RNA was stored at -80°C until use.

cDNA was synthesized using gene-specific primers (Ribobio, Guangzhou, China) and the M-MLV RT kit (Invitrogen, Carlsbad, CA, USA) in a 20μl reaction volume. The RT reaction reagents contained 1mg RNA template, 1μl 10mM dNTP mix, 2μl 0.1 M DTT, 4μl first-strand buffer, and 1μl 40 U/ml RNase inhibitor. The volume was adjusted with RNA-free H2O. The reverse-transcription reaction was performed in triplicate to remove any outliers. MiRNA expression was assessed using qRT-PCR and an ABI PRISM 7500 Sequence Detection System (Applied Biosystems, Foster City, CA). The fold changes in miRNA expression were determined using the 2−ΔΔCT method [10] with the U6 small nuclear RNA (U6) as the reference gene to normalize the data. In addition, we set the expression value of human uroepithelial cell line (SV-HUC-1) as 1 and the expression levels of all the other samples were equal to the relative ratio.

### Statistical analysis

The SPSS (Statistical Package for the Social Sciences) software package, version 18.0 (Chicago, IL, USA), and MedCalc 9.3.9.0 (MedCalc, Mariakerke, Belgium) software were used to analyze all the data. We first used the Kolmogorov-Smirnov test to determine the distribution of the data in each group. The data are presented as the mean ± standard deviation (SD) or median (interquartile range) when the values were normally or abnormally distributed, respectively. Statistical differences between the groups were tested using the Mann-Whitney U-test, Wilcoxon t-test, Student’s t-test, or a Kruskal-Wallis test, as appropriate. Receiver operating characteristic (ROC) curves were established to discriminate the subjects with or without BC. Differences were considered statistically significant only when P<0.05. Multiple-comparisons were considered statistically significant when P values were less than 0.05/(number of contrasts performed) according to the Bonferroni multiple-comparison test.

## Results

### MiR-210 expression in human BC tissues and BC cell lines

**Fig 1:**
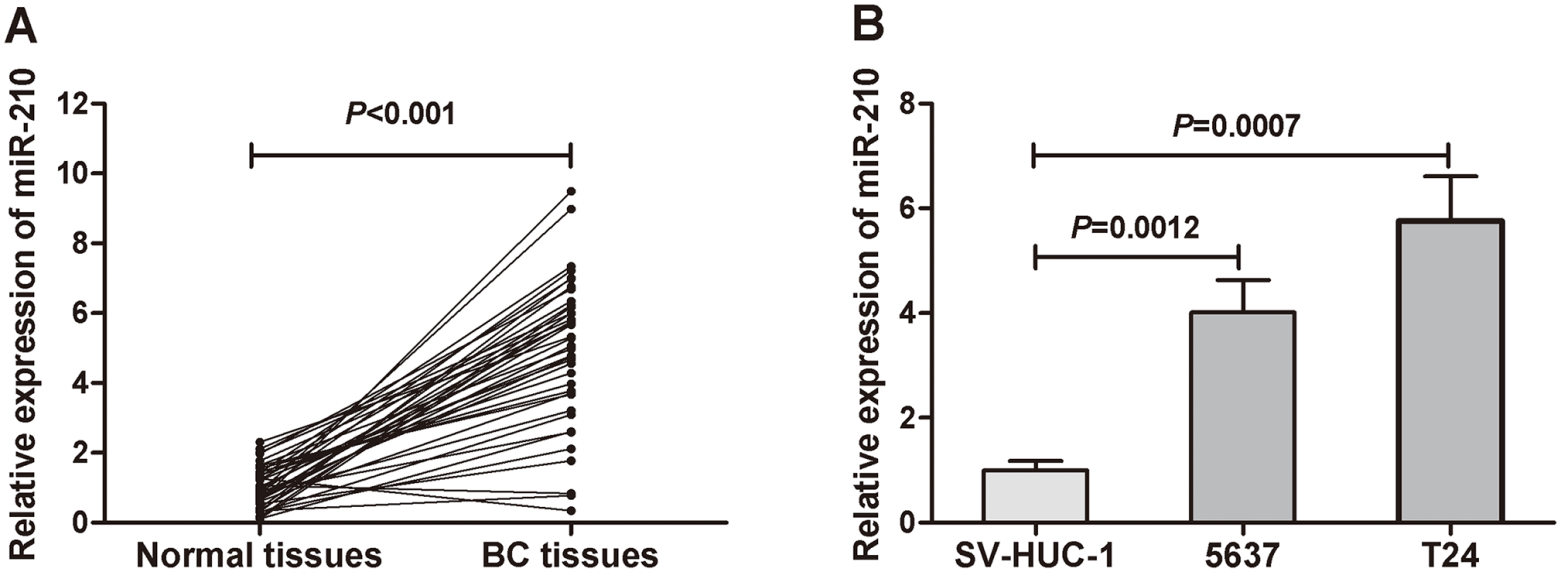
The miR-210 expression in primary BC tissues and BC cell lines. (A) Comparison of miR-210 levels in 40 pairs of primary BC tissues and adjacent normal tissues. The expression level of miR-210 was significant higher in primary BC tissues than in adjacent normal tissues (*P*<0.001). (B) The expression levels of miR-210 in BC cell lines were higher than in a human uroepithelial cell line.

To confirm previously reported high miR-210 expression levels in BC tissues[25], we examined miR-210 expressions in 40 pairs of human BC tissues and corresponding non-cancerous tissues using qRT–PCR. The results showed that the miR-210 expression in the BC tissues was significantly upregulated in comparison to that in adjacent normal tissues (P<0.001, Fig 1A). Also, we investigated the miR-210 expression in human BC cell lines (T24 and 5637) and a human uroepithelial cell line (SV-HUC-1). The results showed that the expression levels of miR-210 in BC cell lines were significantly higher than that in the human uroepithelial cell line (Fig 1B).

### MiR-210 expression in serum of patients with BC

**Fig 2:**
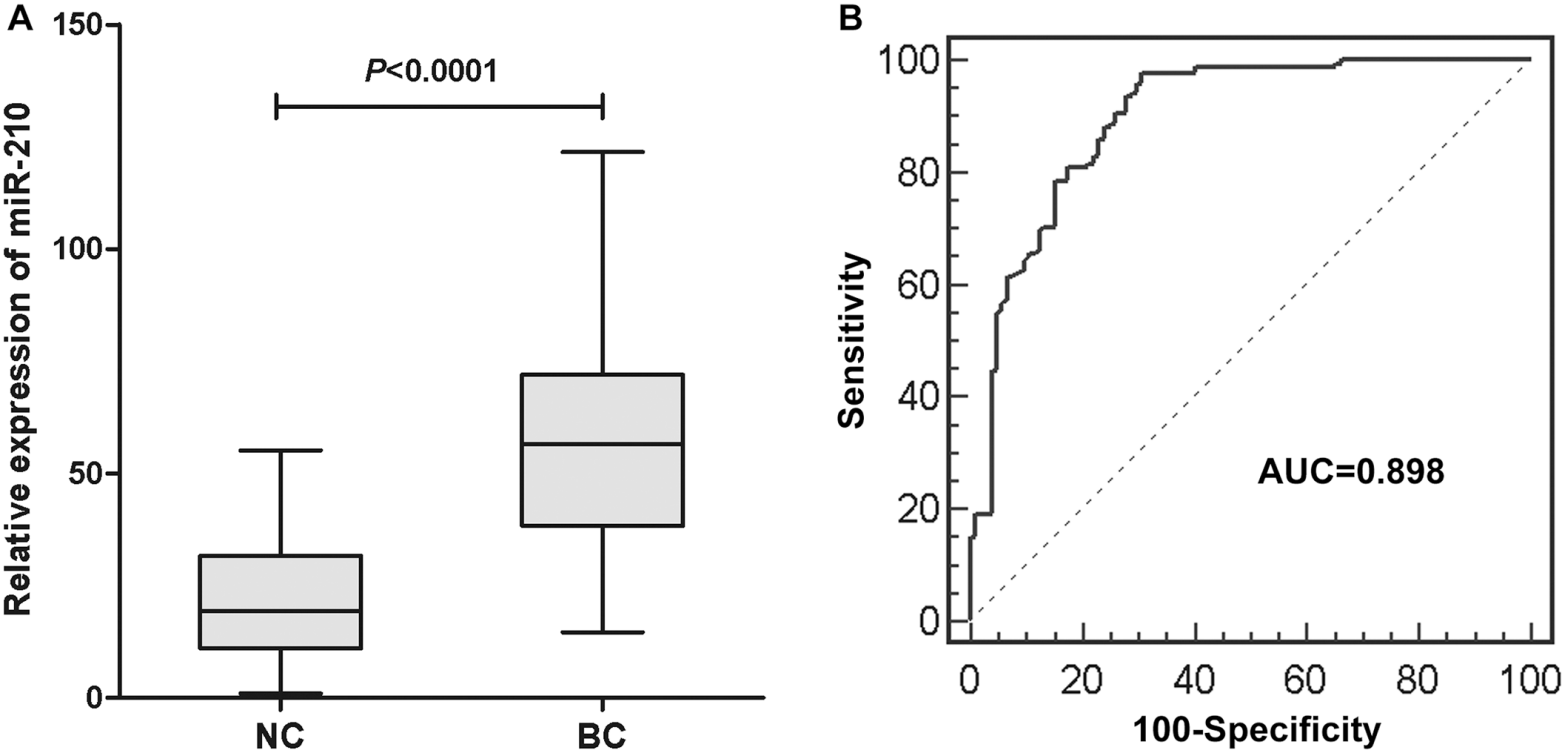
(A) The serum miR-210 expression in 168 BC patients and 104 controls. The upper and lower limits of the boxes and the lines inside the boxes indicate the 75th and 25th percentiles and the median, respectively. The upper and lower horizontal bars denote the 90th and 10th percentiles, respectively. (B) Receiver-operating characteristic (ROC) curve analysis of the serum miR-210 to detect BC patients. The AUC of 0.898 (95% CI 0.855–0.931) indicates good discriminative power.

The expression levels of serum miR-210 in 168 BC patients and 104 controls were examined using qRT-PCR. In order to validate the stability of the reference genes, we compared the expression levels of miR-16-5p, miR-193a-5p and combination of miR-16-5p and miR-193a-5p (geometric mean) in BC patients and normal controls. There was no evidence for differential expression of miR-16-5p, miR-193a-5p and combination of miR-16-5p and miR-193a-5p between BC patients and healthy controls (S1 Fig). And the results indicated that the levels of miR-210 were significantly upregulated in BC patients compared to healthy controls (P<0.0001, Fig 2A). Representation of the data using a ROC plot showed strong separation between both groups, with an AUC of 0.898 (95% CI, 0.855–0.931) (Fig 2B). We used ROC curves with the Younden index to detect cutoff values that could discriminate bladder cancer patients from normal controls [26]. The optimal cutoff value was indicated at 22.37, with a sensitivity of 97.6% (95% CI, 94.0%-99.3%) and a specificity of 69.2% (95% CI, 59.4%-77.9%).

The statistical results showed that serum miR-210 expression in BC patients was significantly correlated with T stage (P = 0.000) and M stage (P = 0.040). There were no significant associations between serum miR-210 expression and the patient’s gender, age, N stage or Grade (all P >0.05) (Table 1).

**Table 1:** MiR-210 expression and clinicaopathological feature in BC patients [median (interquartile range)].

Clinicopathological feature	NO. of cases	miR-210 expression	*P*
Gender			0.475^a^
Male	128	58.680 (39.110–72.473)	
Female	40	54.360 (31.440–89.780)	
Age^c^			0.438^a^
Under 65	74	57.260 (39.350–74.128)	
65 or more	94	56.990 (37.043–72.263)	
Grade			0.058^b^
G1	48	50.620 (31.513–69.203)	
G2	50	54.785 (37.048–69.523)	
G3	70	61.890 (42.170–76.770)	
T stage			0.000^a^
Ta–T1	84	45.805(30.023–60.478)	
T2–T4	84	69.025 (54.336–85.915)	
N stage			0.081^a^
N0	146	56.435 (38.378–69.780)	
N1-3	22	72.315 (37.613–116.763)	
M stage			0.040^a^
M0	154	56.285 (38.298–71.558)	
M1-3	14	71.390 (58.233–88.020)	

^a^Statistical significance was determined by the Kruskal-Wallis test.

^b^Statistical significance was determined by the Mann-Whitey U test.

^c^ The mean age of the patients is 64.8.

### The role of serum miR-210 in predicting tumor stage in patients with BC

**Fig 3:**
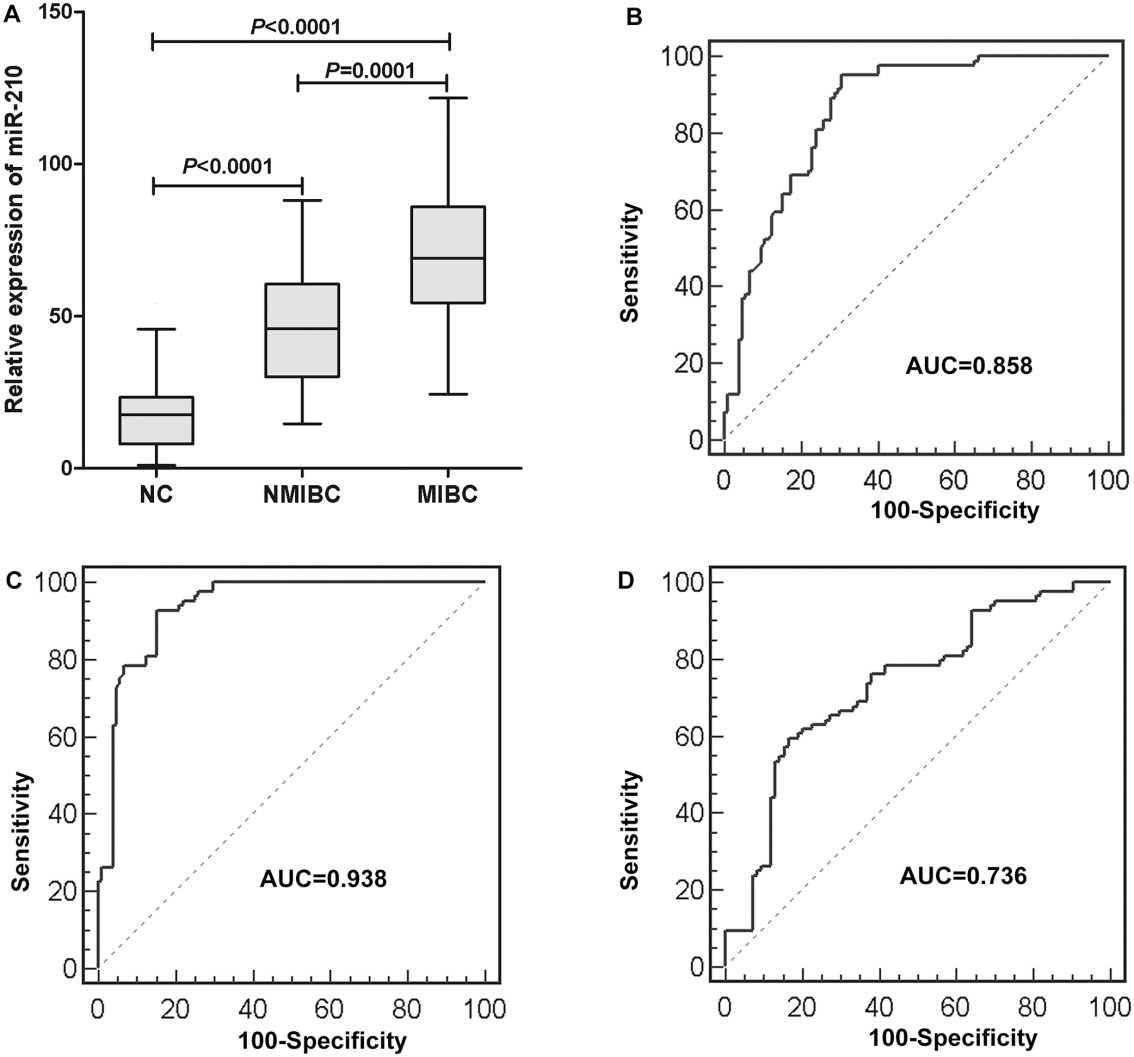
(A) The serum miR-210 expression in 84 NMIBC, 84 MIBC and 104 controls. All *P*<0.05/3. (B) ROC curves for serum miR-210 for NMIBC group (n = 84) vs healthy individuals (n = 104). AUC = 0.858 (95% CI 0.800–0.904). (C) ROC curves for serum miR-210 for MIBC group (n = 84) vs healthy individuals (n = 104). AUC = 0.938 (95% CI 0.893–0.968). (D) ROC curves for serum miR-210 for NMIBC group (n = 84) vs MIBC group (n = 84). AUC = 0.736 (95% CI 0.662–0.800).

In further analysis, we divided the BC patients into NMIBC and MIBC, and found that the serum miR-210 from both NMIBC and MIBC were significantly upregulated compared to the controls (*P*<0.0001 and *P*<0.0001, respectively). More importantly, a significant difference in serum expression of miR-210 was observed between both groups of patients with NMIBC and MIBC (*P*<0.0001, Fig 3A). The AUC of miR-210 used to distinguish NMIBC (Ta–T1) and MIBC (T2–T4) patients from the controls were 0.858 (95% CI, 0.800–0.904) and 0.938 (95% CI, 0.893–0.968), respectively (Fig 3B and 3C). And serum miR-210 could reliably differentiate NMIBC patients from MIBC patients, as evidenced by an AUC value of 0.736 (95% CI, 0.662–0.800) (Fig 3D).

### Evaluation of whether serum miR-210 could monitor tumor dynamics

**Fig 4:**
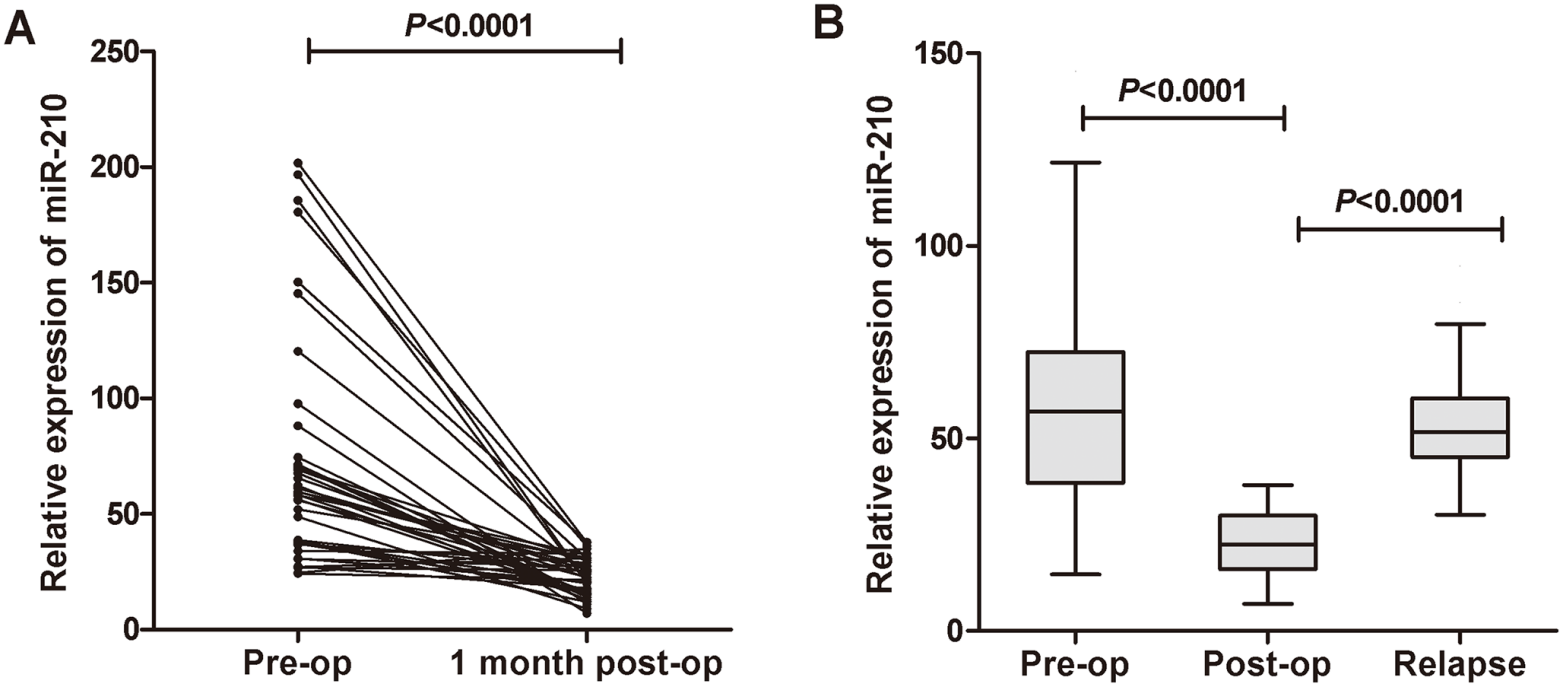
Comparison of circulating miR-210 expressions in different groups. (A) Comparison of serum miR-210 expressions between pre- and post-operative samples from BC patients. The serum miR-210 levels were significantly reduced in paired post-operative samples compared with those in pre-operative samples (*P*<0.0001). (B) Scatter plot of miR-210 levels in the pre-operative (n = 168), post-operative (n = 40) and relapse (n = 30) serum groups.

We selected 10 BC patients with the highest serum miR-210 expression (high group) and another 10 BC patients with the lowest serum miR-210 expression (low group). The corresponding FFPE tissues of these patients were collected and then the miR-210 expressions were evaluated. As a result, in 9 patients of the high group, miR-210 expression in BC FFPE tissues showed higher (90%) than that in patients of the low group (S2 Fig). Moreover, we examined the circulating miR-210 expression levels in paired pre- and post-operative serum samples from 40 BC patients who underwent curative cystectomy, and found that the levels of serum miR-210 were significantly reduced in the post-operative group (P<0.0001) (Fig 4A). We also examined the serum levels of miR-210 in unpaired pre-operative (n = 168), post-operative (n = 40), and relapse (n = 30) serum samples, and found that the miR-210 level was significantly reduced in the post-operative group compared with that in the pre-operative group (P<0.0001). The level of serum miR-210 in the relapse group was significantly increased (P<0.0001) and exhibited a similar level to that in the pre-operative samples (Fig 4B). Taken together, the findings demonstrated that the circulating levels of miR-210 could reflect the tumor dynamics in BC patients.

### Evaluation of monitoring tumor dynamics using miR-210 expression of cultured medium in BC cell lines

**Fig 5:**
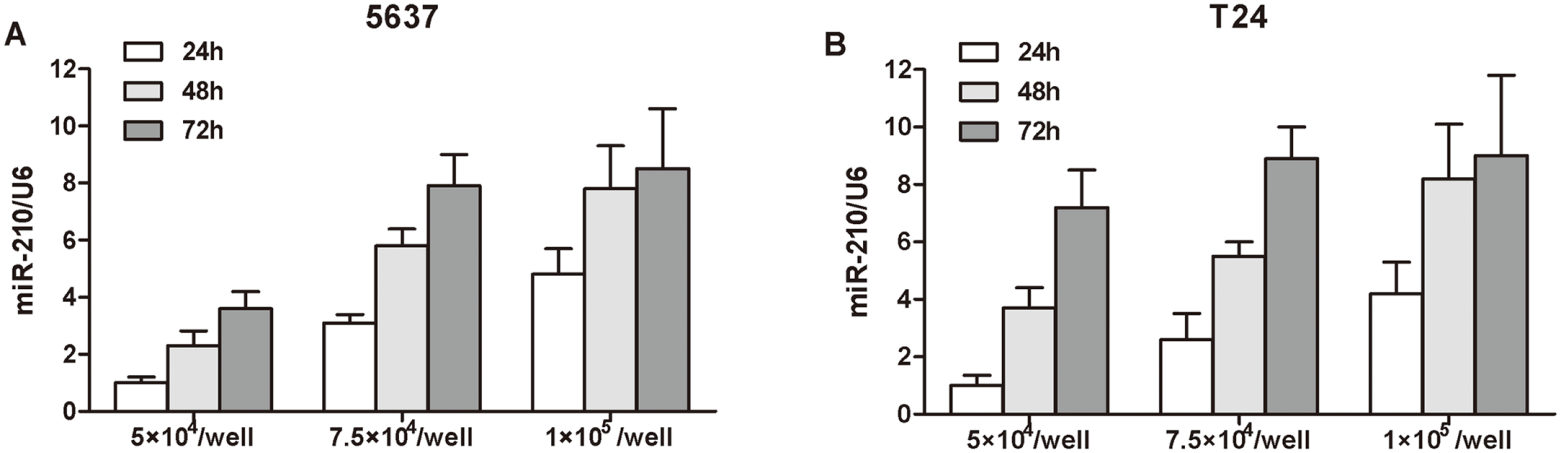
Comparison of medium miR-210 level in BC cells (5637 and T24). Increased tendencies were confirmed in cell medium in both cell number- and time-course-dependent manners.

It has been reported that primary tumor cells could secrete miRNAs into surrounding environments, including adjacent cells and the blood flow [27]. Therefore, we hypothesized that cultured cancer cells might also secrete several cancer-associated miRNAs into surrounding medium, simulating the mutual influence between primary cancer cells and the adjacent blood. To confirm the hypothesis, we first examined the miR-210 expression of cultured medium in BC cell lines (5637 and T24) and found miR-210 were expressed in cultured medium of both 5637 and T24 cells. Then we plated 5637 and T24 cells with different numbers and collected the medium at various time points for detailed investigation. As a result, increased tendencies of the miR-210 expression were confirmed in both cell number- and time-course-dependent manners (Fig 5). These results indicate that miR-210 might be released from primary bladder tumor cells into the surrounding environment, implying that the serum miR-210 might originate from primary BC cells and its serum expression level could reflect tumor dynamics of cancer patients with BC.

## Discussion

Many genetic and epigenetic alterations are found to be involved in tumorigenesis and the progression of various cancers. Previous reports have identified tumor specific changes to nucleic acids in blood of patients, and have showed the potential value of plasma/serum nucleic acids as new non-invasive biomarkers in patients with cancers [28, 29]. Particularly, microRNAs originating from cancer cells have been proven to be stable and easily detected in plasma or serum of patients with several cancers, which could mirror the expression pattern in the neoplastic tissues [10, 11]. But in fact, only relatively few miRNAs that are abundantly expressed in cancer cells are detectable in circulation [30], and nearly 30% of the released miRNAs do not mirror the expression patterns of the specific cancer cells [31]. These reports underscore the importance of finding novel circulating microRNAs as biomarkers for cancers.

MiR-210 is the most consistently and robustly induced microRNA under hypoxia and generally exhibits oncogenic properties in various tumors [32, 33]. Hypoxia is a common feature of many solid tumors, including BC, and is implicated in the regulation of many genes. Recent studies demonstrated miR-210 was upregulated in BC tissues [19, 20]. In this study, first, we confirmed the higher miR-210 expressions in BC tissues and cell lines than in adjacent normal tissues and a human uroepithelial cell line. Then, we focused on the clinical significance of circulating miR-210 in BC. We found that it was significantly higher in BC patients than in healthy volunteers, and the AUC to discriminate BC from healthy controls was 0.898, with a sensitivity of 97.6% and a specificity of 69.2%. This study is the first to demonstrate the potential role of serum miR-210 in detection of BC. We also found that high miR-210 expression in serum was associated with advanced T stage and the presence of metastasis. These findings were consistent with other reports that high miR-210 expression in BC tissue was associated with muscle-invasive aggressiveness [19, 20].

Further research showed miR-210 levels could not only distinguish NMIBC patients from healthy control patients, but also MIBC from NMIBC with AUC value of 0.736. However, the relatively low AUC and the considerable overlap in distributions of serum miR-210 in NMIBC and MIBC indicate that serum miR-210 could not be used to accurately differentiate between MIBC and NMIBC. Nevertheless, serum miR-210 may serve to assess the relative likelihood of muscle-invasive BC in the absence of more reliable circulating biomarkers. Thus, it could be helpful in preoperatively predicting tumor stage with the hope that such knowledge could possibly guide therapeutic choice.

We also investigated whether serum miR-210 expression levels could reflect tumor dynamics in BC. The comparison between miR-210 expression in paired serum and primary tumors revealed that high levels of miR-210 represented higher expressions in most cases of primary BC tissues. miR-210 levels were also compared in paired serum samples obtained before and after curative surgery, which exhibited significantly reduction postoperatively. And the levels of serum miR-210 from patients with relapsed BC were upregulated and reached the levels of that in pre-operative patients. Moreover, we determined whether the miR-210 level in culture medium could reflect the status of BC cell lines and might be released from cancer cells. And our data clearly demonstrated that the miR-210 levels in cultured medium of BC cell lines significantly increased in both cell number- and time-course-dependent manners, while the miR-210 levels in cultured medium of the human uroepithelial cell line remained unchanged (data not shown). These findings indicated that the serum expression level of miR-210 could be reflective of tumour dynamics and might monitor tumor status.

Although our current assay may be potentially useful for BC detection, there was a limitation of using miR-210 as a single biomarker for the diagnosis of BC. Circulating expression of miR-210 has also been described in other solid cancers, such as breast cancers[22], kidney cancer [34], and pancreatic cancer [35]. Therefore, it might be a challenge to discriminate whether high circulating miR-210 level is specifically associated with BC itself or if this is only a common phenomenon which manifests as a result of perturbations in the host immune response during progression of any cancer [36]. We addressed this issue by demonstrating that a positive significant correlation existed between primary tumor tissue and serum levels of miR-210 and that serum miR-210 levels fell postoperatively in a subset of patients, highlighting the specificity of miR-210 as a specific biomarker for BC.

In conclusion, our results provide compelling evidence that serum miR-210 could be used as a noninvasive biomarker for screening, stage predicting and monitoring BC. This concept can be incorporated into clinical decisions in the not-so-distant future pending validation in large-scale prospective trials.

## Supporting Information

S1 Fig Ct values of miR-16-5p, miR-193a-5p, combination of miR-16-5p and miR-193a-5p in BC patients and healthy controls (all *P*>0.05). No significant difference was found within 3 reference genes in both groups.
(TIF)

S2 Fig Comparison of miR-210 levels in serum and corresponding cancer tissues. (TIF)
