# Supplementary figures and images for: Serum miR-210 Contributes to Tumor Detection, Stage Prediction and Dynamic Surveillance in Patients with Bladder Cancer
Source: PLoS One. 2015 Aug 7;10(8):e0135168. doi: 10.1371/journal.pone.0135168 (PMC4529273; doi:10.1371/journal.pone.0135168)

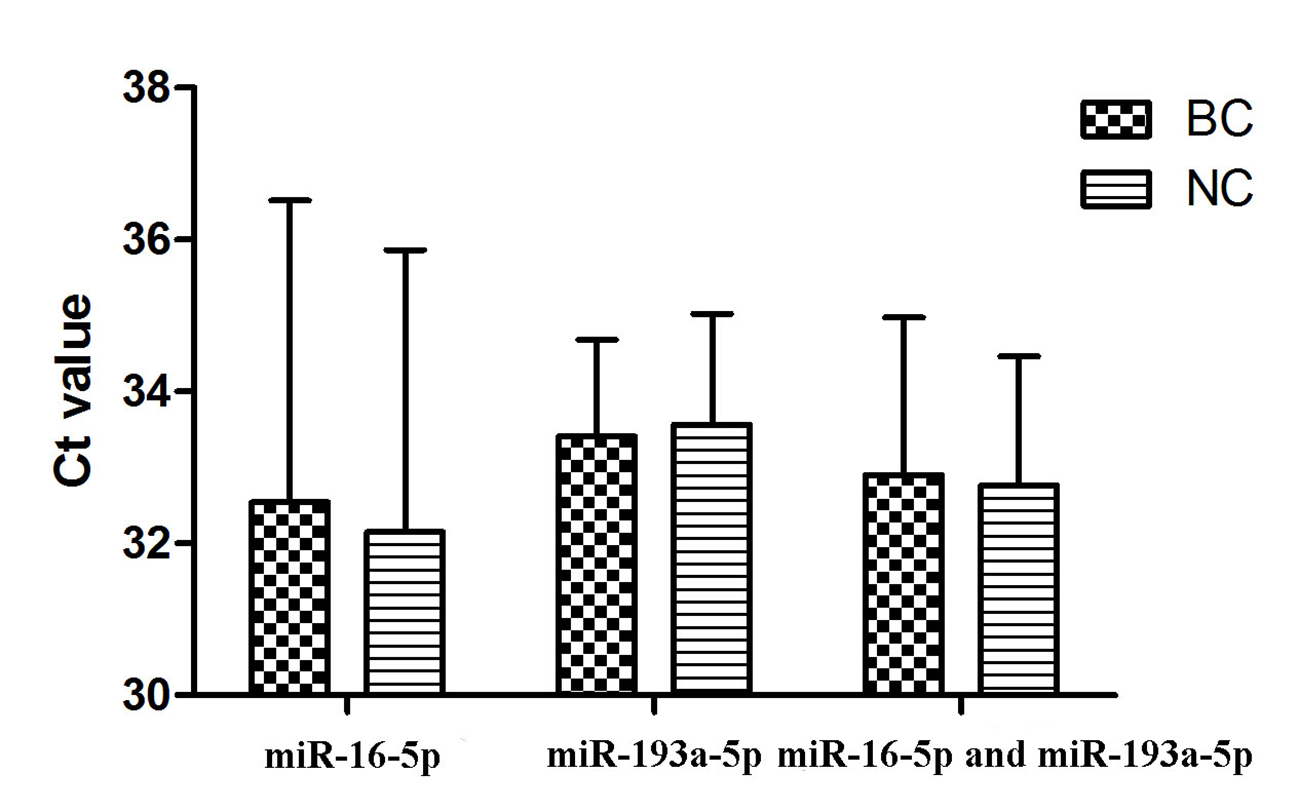

Supplement: S1 Fig — No significant difference was found within 3 reference genes in both groups. (TIF) [file pone.0135168.s001.tif]

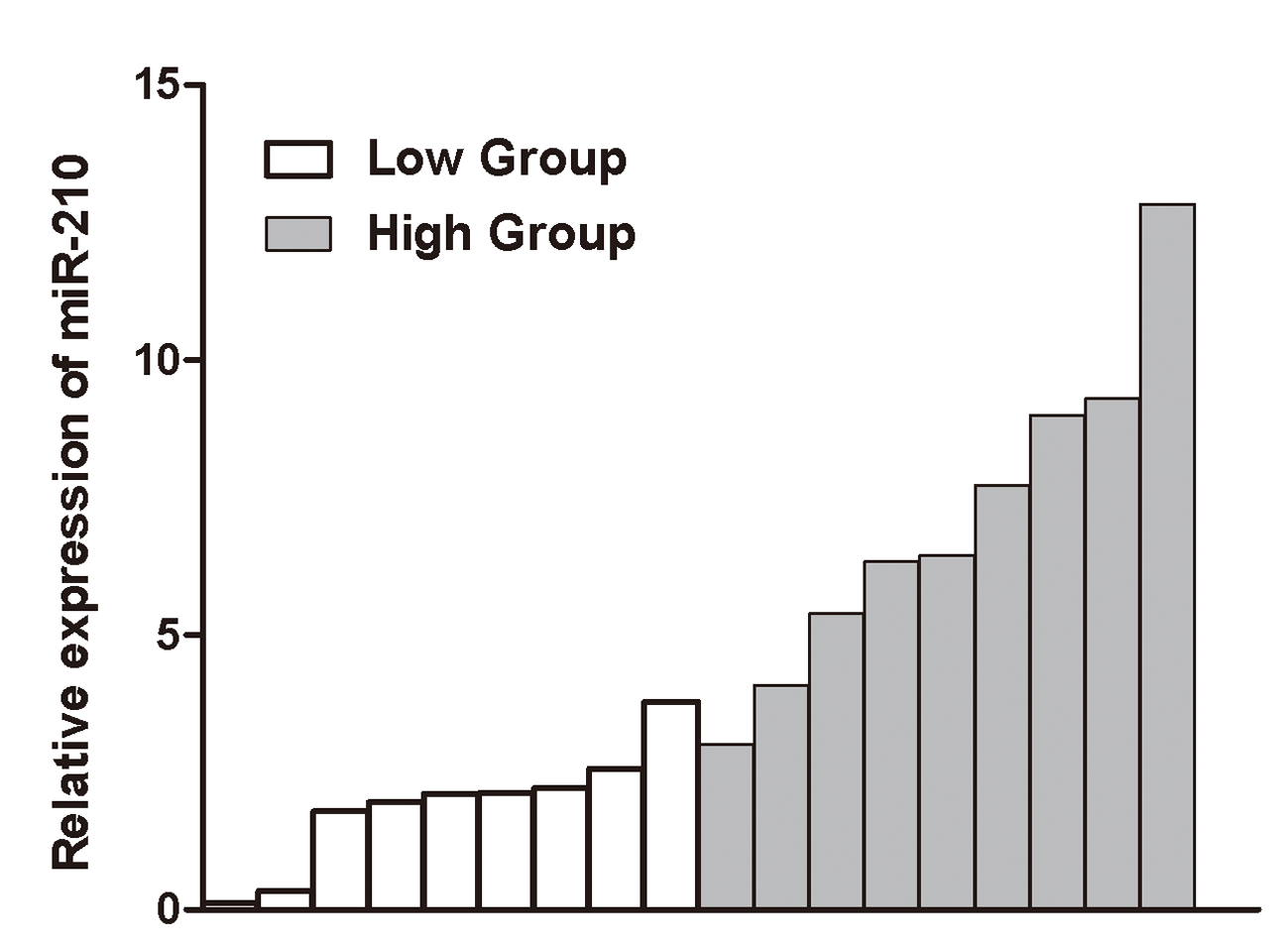

Supplement: S2 Fig — (TIF) [file pone.0135168.s002.tif]
